# Vpx-Independent Lentiviral Transduction and shRNA-Mediated Protein Knock-Down in Monocyte-Derived Dendritic Cells

**DOI:** 10.1371/journal.pone.0133651

**Published:** 2015-07-24

**Authors:** Wojciech Witkowski, Jolien Vermeire, Alessia Landi, Evelien Naessens, Hanne Vanderstraeten, Hans Nauwynck, Herman Favoreel, Bruno Verhasselt

**Affiliations:** 1 Department of Clinical Chemistry, Microbiology and Immunology, Ghent University, De Pintelaan 185, Gent, Belgium; 2 Virology lab, Department of Virology, Parasitology and Immunology, Ghent University, Salisburylaan 133 D1, Merelbeke, Belgium; 3 Immunology lab, Department of Virology, Parasitology and Immunology, Ghent University, Salisburylaan 133 D1, Merelbeke, Belgium; Jackson Laboratory, UNITED STATES

## Abstract

The function of dendritic cells (DCs) in the immune system is based on their ability to sense and present foreign antigens. Powerful tools to research DC function and to apply in cell-based immunotherapy are either silencing or overexpression of genes achieved by lentiviral transduction. To date, efficient lentiviral transduction of DCs or their monocyte derived counterparts (MDDCs) required high multiplicity of infection (MOI) or the exposure to the HIV-2/SIV protein Vpx to degrade viral restriction factor SAM domain and HD domain-containing protein 1 (SAMHD1). Here we present a Vpx-independent method for efficient (>95%) transduction of MDDCs at lower MOI. The protocol can be used both for ectopic gene expression and knock-down. Introducing shRNA targeting viral entry receptor CD4 and restriction factor SAMHD1 into MDDCs resulted in down-regulation of targeted proteins and, consequently, expected impact on HIV infection. This protocol for MDDCs transduction is robust and free of the potential risk arising from the use of Vpx which creates a virus infection-prone environment, potentially dangerous in clinical setting.

## Introduction

Dendritic cells (DCs) are crucial actors in the interplay between pathogens and the immune system, linking innate and adaptive immune responses. DCs capture incoming pathogens and present them to T cells [1]. Their important role in induction of anti-tumor immunological responses raises hope that use of this potential will lead to efficient cell-based immunotherapy [2]. Understanding mechanisms that shape DC crosstalk between immunogens and components of the immune system, is a prerequisite for successful clinical implementation of such therapeutic approach, both in oncology and infectious diseases. Research is hampered by the difficulties to manipulate DCs gene expression profile, especially when it comes to reduction of gene expression. Selective knock-down of gene products by RNA interference is a widely used method in the study of gene function [3]. There are several methods of triggering RNA interference into the cells with small interfering RNA (siRNA) and short hairpin RNA (shRNA) being the most commonly applied. In our study we chose lentivirus-mediated shRNA expression as it provides stable knock-down levels, while producing fewer off-target effects than transfection-based siRNA delivery [4, 5]. Lentiviral transduction of monocyte-derived DCs (MDDCs) has been described, however transduction efficiency was quite low (<40% of transduced cells) [6] or required very high dose of vectors (multiplicity of infection (MOI) of 150) which led to up-regulation of maturation markers [7]. For DC therapy, efficient gene transfer to express antigen is highly desired. Transduction efficiency at lower vector MOI could be increased by careful timing, spinoculation and by use of agents such as polybrene. Despite this, 90% efficiency was rarely attained [8]. The HIV-2/SIV protein Vpx was found to ameliorate the transduction level up to 10-fold [9]. Consequently, this observation led to the discovery of an important cellular lentivirus resistance factor: SAM domain and HD domain-containing protein 1 (SAMHD1) [10, 11], targeted by Vpx. SAMHD1 combines the ability to deplete the cytoplasmic pool of dNTPs necessary for the reverse transcription of viral genome [12] together with ribonuclease activity which degrades incoming viral RNA [13], thereby highly decreasing the chances of successful lentiviral integration. Vpx is known to target SAMHD1 for proteasomal degradation [10, 11]. Exposure to Vpx loaded virus-like particles as a method to overcome SAMHD1 restriction might confer the DCs with distinct features which can affect the read-out of the genetic manipulation intended by the transduction. A critical phenotypic alteration of DCs induced by Vpx-induced SAMHD1 block is their subsequent permissiveness to viral infections–a caveat for clinical applications. To circumvent this, we developed a method for efficient Vpx-independent lentiviral transduction, which allows lentivirus-based shRNA delivery to MDDCs at high efficiency (>95%). Using SAMHD1 as a target, we show effective gene knock-down at the protein level resulting in enhanced HIV infection of the transduced cells. This method preserves the immature MDDC phenotype, which makes it an important tool in studies of DC function and differentiation.

## Materials and Methods

### Ethics statement

The study protocol was approved by the Ghent University Hospital Ethical Committee. Donor samples were obtained after informed consent.

### Monocyte isolation

Monocytes were isolated from buffy coats of healthy donors following Lymphoprep (Axis-Shield, Dundee, Scotland) gradient centrifugation and positive or negative magnetic antibody separation kit (Miltenyi Biotec, Leiden, Netherlands). Purity was assessed by flow cytometry of anti-CD14-PE stained cells and was always found to be above 95%. Isolated cells were cultured in 24-well plates at 250 000 cells/well in 0.5 mL of RPMI medium (RPMI 1640, Life Technologies, Carlsbad, CA) supplemented with 2 mM L-glutamine (Life Technologies), 2.5% (vol/vol) heat inactivated fetal calf serum (FCS, Hyclone Perbio, Thermo Scientific, Rockford, IL), 100 U/mL penicillin, 100 μg/mL streptomycin (Life Technologies), IL-4 at 500 IU/mL and GM-CSF at 1 000 IU/mL (Gentaur, Kampenhout, Belgium) at 37°C in a humidified atmosphere containing 5% (vol/vol) CO_2_. For the assessment of the impact of fetal calf serum on MDDC transduction efficiency, sera from Biochrom (Merc Milipore, Overijse, Belgium), Bovogen Biologicals (East Keilor, Australia), Lonza (Verviers, Belgium) and PAA (GE Healthcare, Diegem, Belgium) were used additionally.

### Titration of lentiviruses

To ensure standardized transduction, lentiviral supernatants should be titrated. Timely biological titration assays can be replaced by measurement of viral reverse transcriptase (RT) activity [14]. Supernatant of lentiviral vector encoding a scrambled shRNA sequence and an eGFP marker gene, which showed MOI of 10 when measured on 293T cells, provided over 95% MDDC transduction efficiency. This lentiviral supernatant was found to express RT activity of 5,550 mU/ml (equivalent of 1 μg of p24/ml). Aliquots of that supernatant were included in all subsequent reverse transcriptase activity assays and served as a standard reference for all viral productions.

### Lentiviral transduction

Unless stated otherwise, monocytes obtained by positive magnetic bead-based selection of CD14+ cells were used in experiments. On day 1 post-monocyte isolation, medium was replaced with fresh medium containing 50% lentiviral supernatant (final cytokine concentration unchanged). Typically RT activity of 2 750–5 550 mU/ml was used. Cells were subsequently spinoculated (90 min, 950 g, 32°C) in the presence of polybrene (4 μg/mL; Sigma-Aldrich, Diegem, Belgium). Medium was refreshed 24 h post-transduction and culture in presence of IL-4 and GM-CSF was continued until day 6. In some experiments, maturation was induced with LPS (100 ng/mL; Sigma-Aldrich) as described by Izquierdo-Useros N et al. [15]. From day 6 post-transduction onwards, cells were cultured in 10% FCS (vol/vol) RPMI medium supplemented with glutamine, penicillin and streptomycin. For Vpx assisted transductions, lentiviral supernatants were mixed with Vpx-VLPs (1:1). VLP containing supernatant was used at 5 550 mU RT/ml.

### Production of lentiviral vectors, Vpx Virus Like Particles (VLP) and replication-competent HIV-1 reporter virus

Lentiviral vector production from pLKO.1 vectors in 293T cells was done as reported previously [16]. For SAMHD1 and DC-SIGN down-regulation shRNA clones TRCN0000145408 and TRCN0000029690 respectively (Sigma Aldrich) were used (provided by BCCM/LMBP and the Hercules Foundation, Zwijnaarde, Belgium). For CD4, shRNA clone TRCN0000057616 was used (Sigma Aldrich). Vpx containing VLPs were produced by co-transfecting (JetPei Polyplus, Sélestat, France) 293T cells, according to manufacturer’s instructions, with vesicular stomatitis virus envelope plasmid pMD.G [17] and minimal, self-inactivating SgpΔ2 plasmid, kind gift of Dr. K. Überla (Ruhr-University-Bochum, Germany) [18]. Replication-competent, CCR5 tropic HIV-1 virus was generated by swapping a SalI-BamHI fragment containing most of the *env* gene from the pNL4-3 proviral construct NLENG1-IRES [19] with the envelope from NL4-3-Bal-IRES-HSA [20, 21]. The resulting NL4-3-Bal-IRES-EGFP plasmid was transfected into 293Ts with or without vesicular stomatitis envelope plasmid as described for Vpx VLP production. For all the viral productions, supernatants were refreshed 24 h and collected 48 h after transfection, centrifuged at 900 g for 10 min to pellet the remaining cells and stored at -80°C until use. The titer of the viral supernatants was measured by quantification of reverse transcriptase activity via real time-PCR and expressed as equivalent p24 as described above.

### HIV infection of transduced MDDCs

Six days post-transduction the cells were plated in a 96-well plate at 50 000 cells/well and HIV infected (50 ng p24) by spinoculation (90 min, 950 g, 32°C) in presence of 1 μM ritonavir (NIH AIDS Reagent Program, Germantown, MD) in a final volume of 200 μL. On day 1 post-infection medium was refreshed. Infection was measured on day 3 by flow cytometry, gating on EGFP expressing, live cells as judged by propidium iodide staining (Miltenyi Biotec).

### Antibodies and flow cytometry

Monoclonal mouse anti-human CD4-APC (M-T466), CD80-APC (2D10), CD86-APC (FM95) and CD209-PE (DCN47.5, DC-SIGN) were purchased from Miltenyi Biotec. Monoclonal mouse anti-human CD-14 PE (MφP9) and CD83-PE (HB15e) were purchased from BD Biosciences (San Diego, CA). Polyclonal rabbit anti-human SAMHD1 was purchased from Proteintech (Chicago, IL). Alexa 660 Fluor Goat Anti-Rabbit IgG (H+L) was purchased from Life Technologies. Stained cells were analyzed on MACSQuant (Miltenyi Biotec) or FACS Calibur flow cytometer (BD Biosciences, Erembodegem, Belgium).

### Intracellular SAMHD1 staining

Intracellular staining of SAMHD1 was performed at room temperature in the dark. For fixation, 50 000 cells were incubated for 10 min in fixation medium (AN DER GRUB Bio Research, Wien, Austria) and washed twice (PBS with 1% FCS (v/v) and 0.09% (g/l) sodium azide). Intracellular SAMHD1 staining was performed in permeabilization medium (AN DER GRUB Bio Research) for 30 min, washed twice and stained with goat-anti rabbit IgG in wash buffer. Alternatively, to facilitate antibody access to the nucleus, cells fixed in 4% formaldehyde (Klinipath B.V., Olen, Belgium) were permeabilized with 0.25% Triton X-100 (Sigma-Aldrich) and stained in wash buffer.

### Statistical analysis

Nonparametric Mann-Whitney U test was performed using GraphPad Prism version 5.00 for Windows (GraphPad Software, San Diego, CA).

## Results

### Efficient lentiviral transduction in absence of Vpx

**Fig 1:**
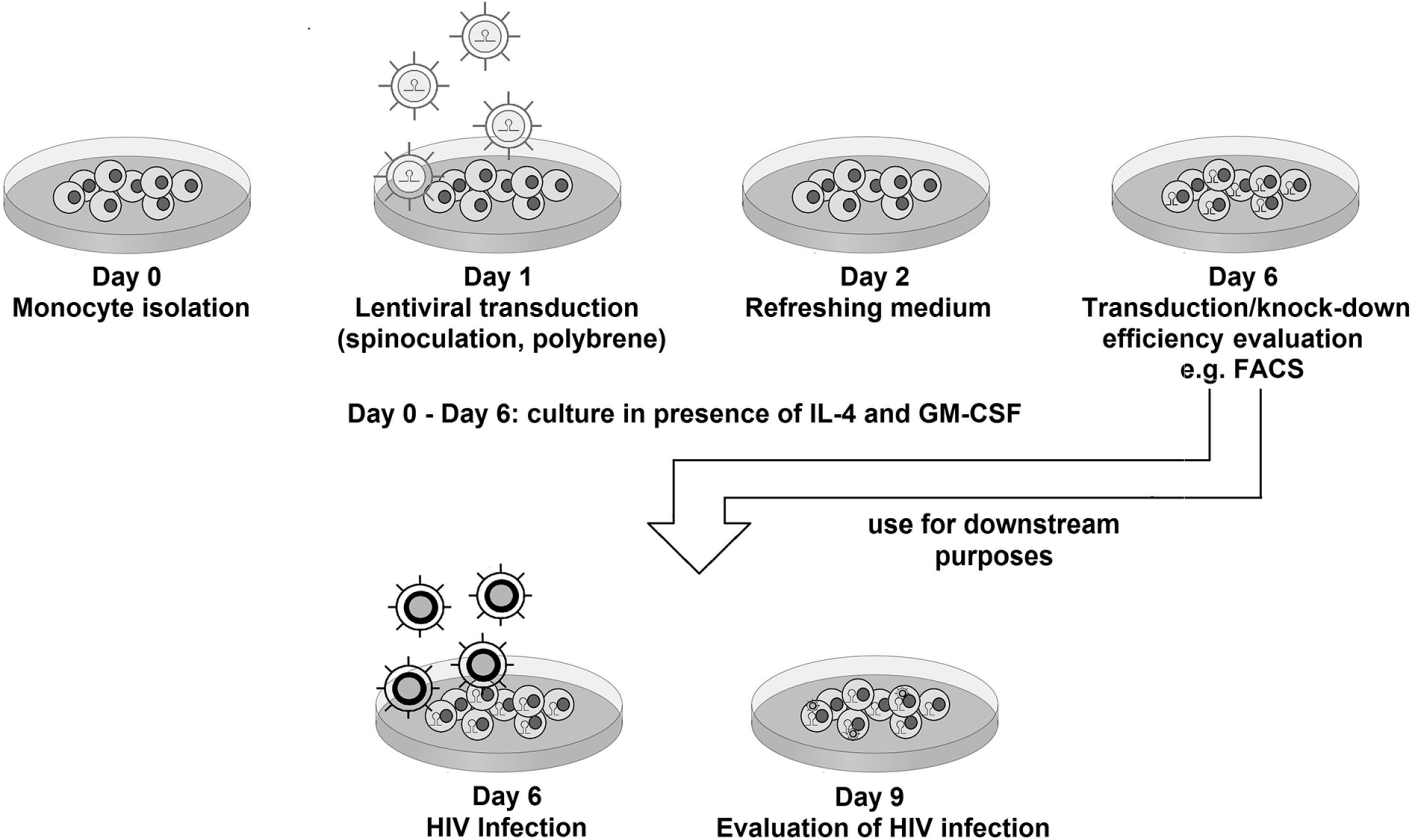
Method overview. Transduction workflow as described in the Materials and Methods section.

**Fig 2:**
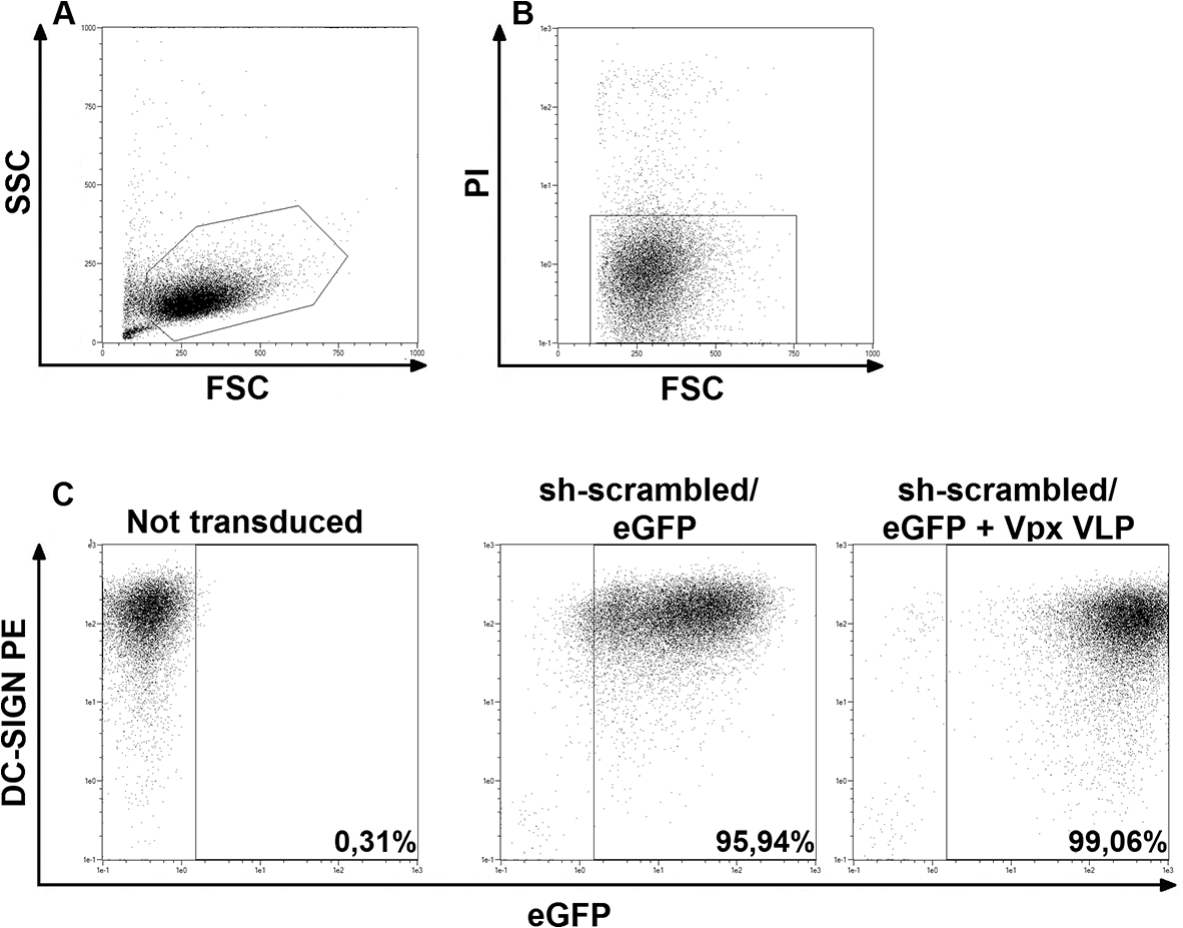
MDDCs can be transduced at high efficiency in the absence of Vpx. Cells were transduced with a lentiviral vector encoding eGFP and a scrambled shRNA sequence 24 hours post-isolation from PBMCs in the absence or presence of Vpx-containing VLP, and analyzed by flow cytometry 6 days post-isolation. The top panel shows MDDC gating strategy. (A) Cells were gated based on their forward scatter (FSC) and side scatter (SSC) profile. (B) Cells gated as in (A) were subsequently gated to exclude dead cells from further analysis by setting a gate on live (propidium iodide (PI)-negative) cells. (C) Bottom dot plots show eGFP expression versus DC-SIGN-PE on MDDCs, transduced or not, as indicated. Percentages indicate fraction of eGFP expressing cells.

We optimized the transduction of MDDCs in the absence of Vpx, by investigating the effect of a range of parameters (additives like polybrene, spinoculation, experimental timeline) to reach the most effective protocol as described in Methods and depicted in Fig 1. In agreement with previous studies [8, 22], timing had the biggest impact on the number of transduced cells. In the final protocol, transduction was performed by spinoculation in presence of polybrene shown to facilitate virus-cell binding and entry [8, 23]. To measure transduction efficiency, we used a pLKO.1-derived lentiviral vector encoding a scrambled shRNA sequence (not targeting any transcript in the human genome) and an eGFP marker gene as described before [14]. Transduction efficiency as well as the MDDC phenotype were evaluated five days post-transduction. As shown in Fig 2, over 95% of cells express the transgene, notably without affecting surface DC-SIGN expression. In comparison, transduction in the presence of Vpx was superior for the expression level of the transgene, although a similar fraction of transduced cells could be reached with our optimized Vpx-independent protocol. Notably, on day 5 post-transduction, when monocytes are differentiated into MDDCs, the number of cells recovered from culture was comparable to that of non-transduced control cells. Efficiency of transduction was highly reproducible among the tested donors (S1 Fig). We observed, that similar transduction efficiency can be achieved regardless of monocyte isolation method (positive vs. negative separation with magnetic beads) as depicted in S2 Fig Identical transduction levels were observed upon culture in media supplemented with fetal calf sera from several manufacturers (S3 Fig), indicating efficient transduction is not the result of the particular serum batch used.

### Transduction of MDDCs does not induce maturation and cells retain the potential to undergo maturation

**Fig 3:**
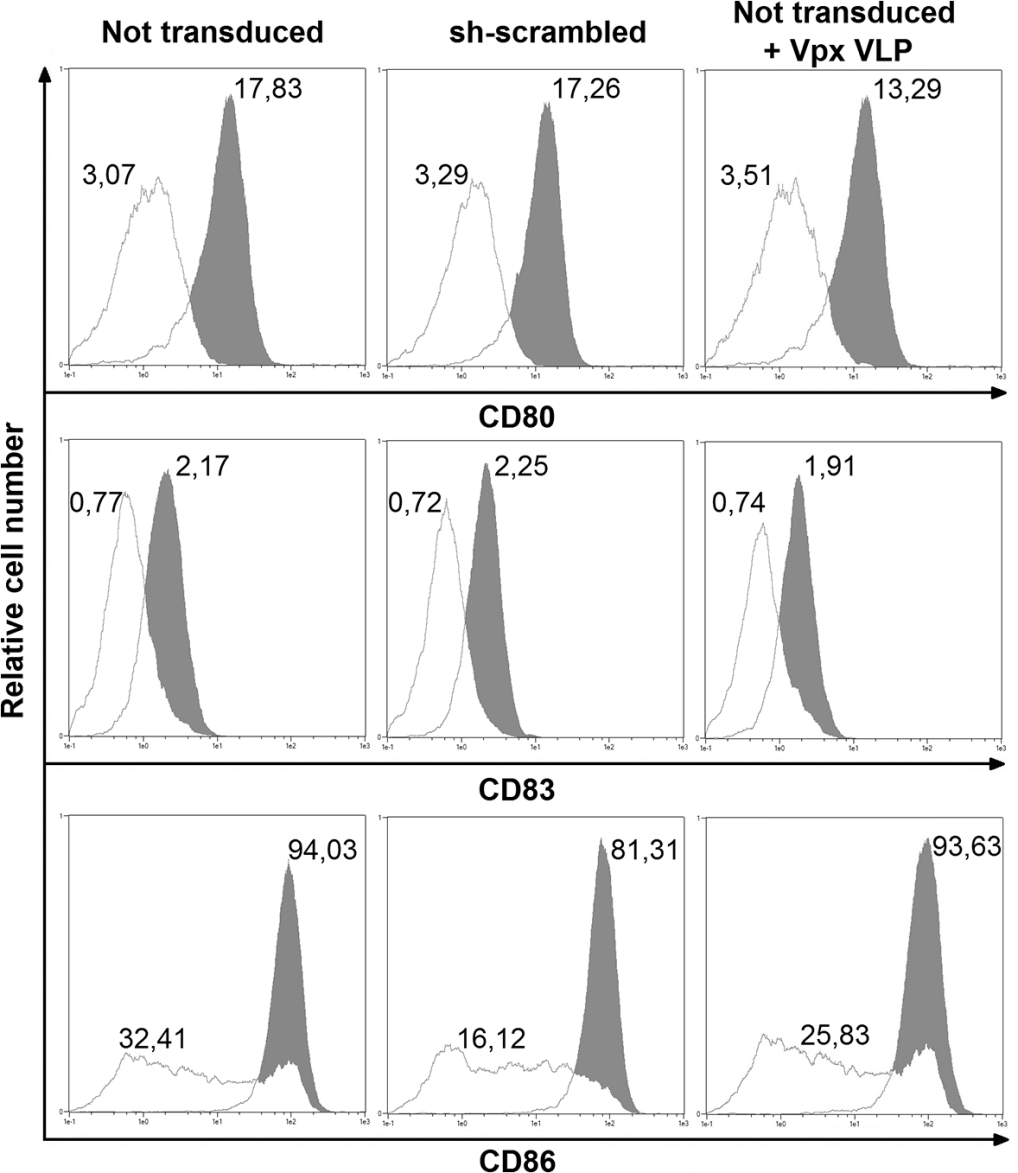
Transduced MDDCs do not mature per se, but retain the potential to undergo maturation. MDDCs were exposed to LPS (filled profiles) or not (open profiles) starting 4 days post-isolation. Histograms show CD80 APC, CD83 PE and CD86 APC surface staining 48h later in control non-transduced, non-transduced but exposed to Vpx VLPor shRNA-scrambled transduced cells as indicated. Numbers represent mean fluorence intensity.

To demonstrate that our method does not induce maturation of MDDCs as other methods previously described [7], yet still allows for it to occur, we measured surface expression of maturation markers on cells cultured in the presence or not of Toll Like Receptor 4 ligand LPS. As shown, CD80, CD83 and CD86 (Fig 3) levels on transduced cells were similar to non-transduced cells as well as cells exposed to the Vpx VLP. Addition of LPS induced the expression of maturation markers.

### Vpx-independent, shRNA-mediated gene knock-down in MDDCs

**Fig 4:**
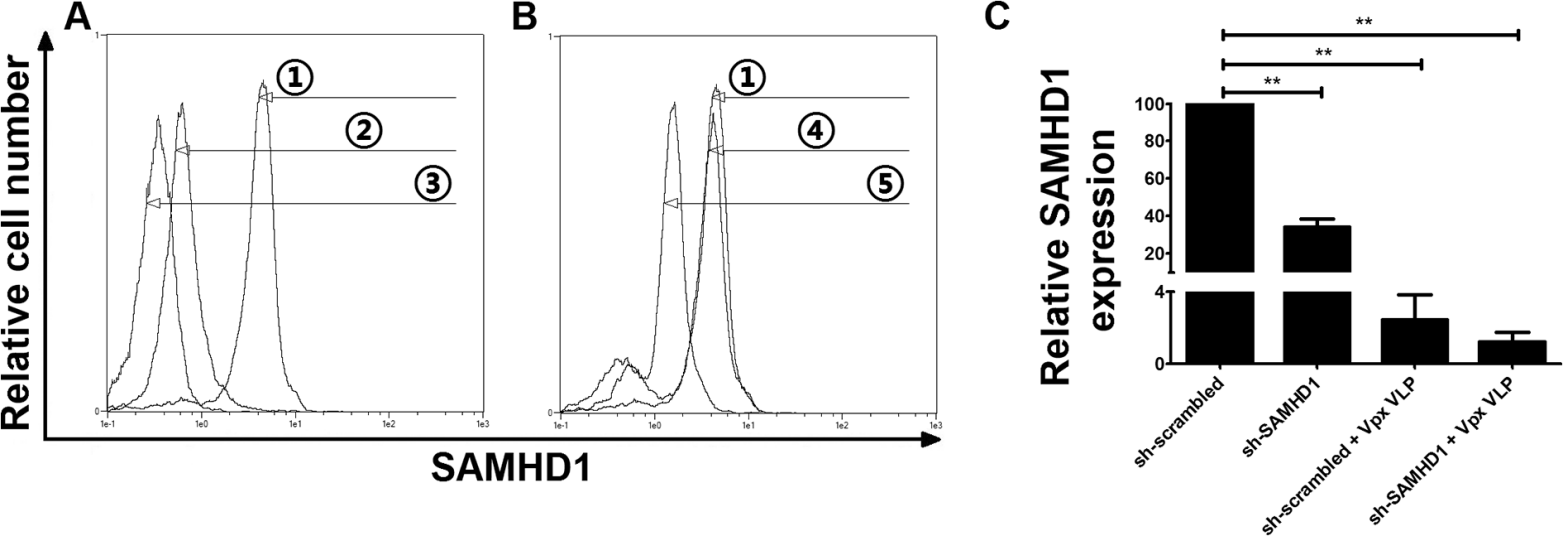
Vpx-independent, shRNA-mediated knock-down of SAMHD1 in MDDCs. (A) Histogram shows intracellular SAMHD1-Alexa 660 staining at similar time point after isolation as shown in B, without ① or with ② treatment with Vpx VLP. Control histogram represents cells stained only with secondary, goat-anti rabbit Alexa 660 antibody ③. (B) Histogram shows intracellular SAMHD1-Alexa 660 staining in MDDCs, 5 days post-transduction with scrambled shRNA (sh-scrambled) ④, or sh-SAMHD1 vectors ⑤ as indicated. Histogram depicting SAMHD1 levels in not transduced cells was overlaid for comparison ①. (C) The bar graph shows SAMHD1 down-regulation in MDDCs from 5 independent donors transduced similar to panel B with or without the addition of Vpx VLP. Error bars represent standard deviation among donors, comparison to sh-scrambled **p ≤ 0.008.

**Fig 5:**
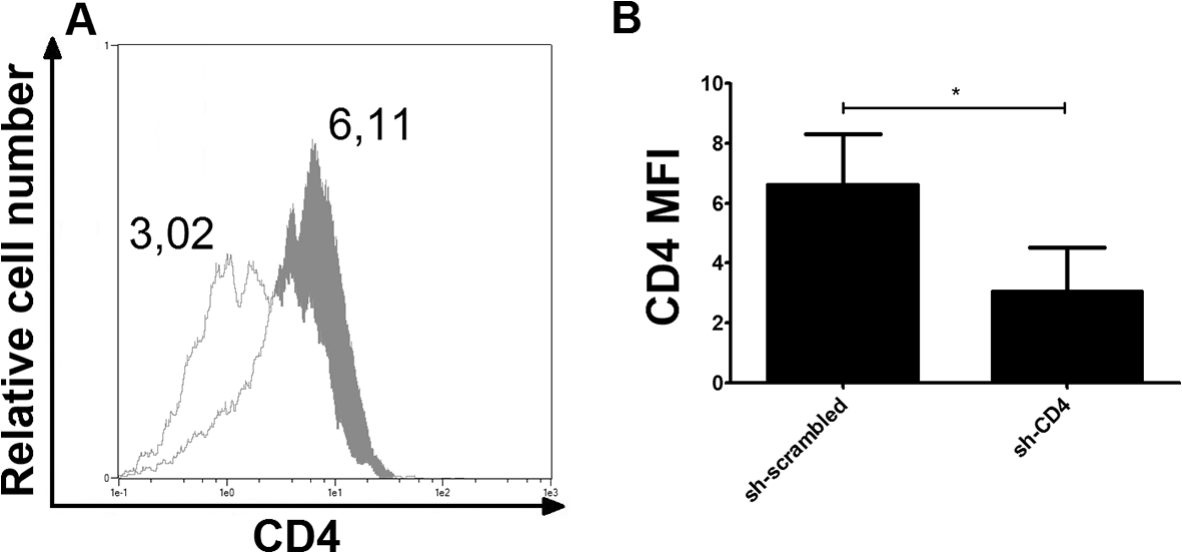
Vpx-independent, shRNA-mediated CD4 down-regulation in MDDCs. (A) Histogram shows CD4 surface expression upon sh-CD4 (open profile) or sh-scrambled transduction on day 5 post-transduction (filled profile) in a representative experiment. Numbers represent mean fluorescence intensity. (B) Bar graph represents mean fluorescence intensity of anti CD4-APC surface staining of MDDCs 5 days post-transduction with respective shRNA constructs. Error bars represent standard deviation among donors *p ≤ 0.028 (N = 4).

Since our method reaches high transduction efficiencies, we wondered if shRNA-mediated knock-down of protein expression in MDDCs was feasible. We choose SAMHD1 and CD4 as targets since this allows us to measure a functional effect of the knock-down on HIV infection. Transduction with pLKO.1 lentiviral vectors encoding an shRNA targeting SAMHD1 clearly reduced the SAMHD1 expression in all donors tested (Fig 4). Notably, knock-down levels were very reproducible among the donors. Expression of SAMHD1 was also greatly reduced by Vpx alone even five days after exposure to the Vpx-loaded Virus Like Particles (VLP)s. We achieved similar, reproducible levels of CD4 down-regulation when CD4 specific shRNA was used (Fig 5). Likewise, an efficient knock-down of dendritic cell-specific intercellular adhesion molecule-3-Grabbing non-integrin protein (DC-SIGN) was observed (S4 Fig).

### HIV infection of lentivirus-transduced cells

**Fig 6:**
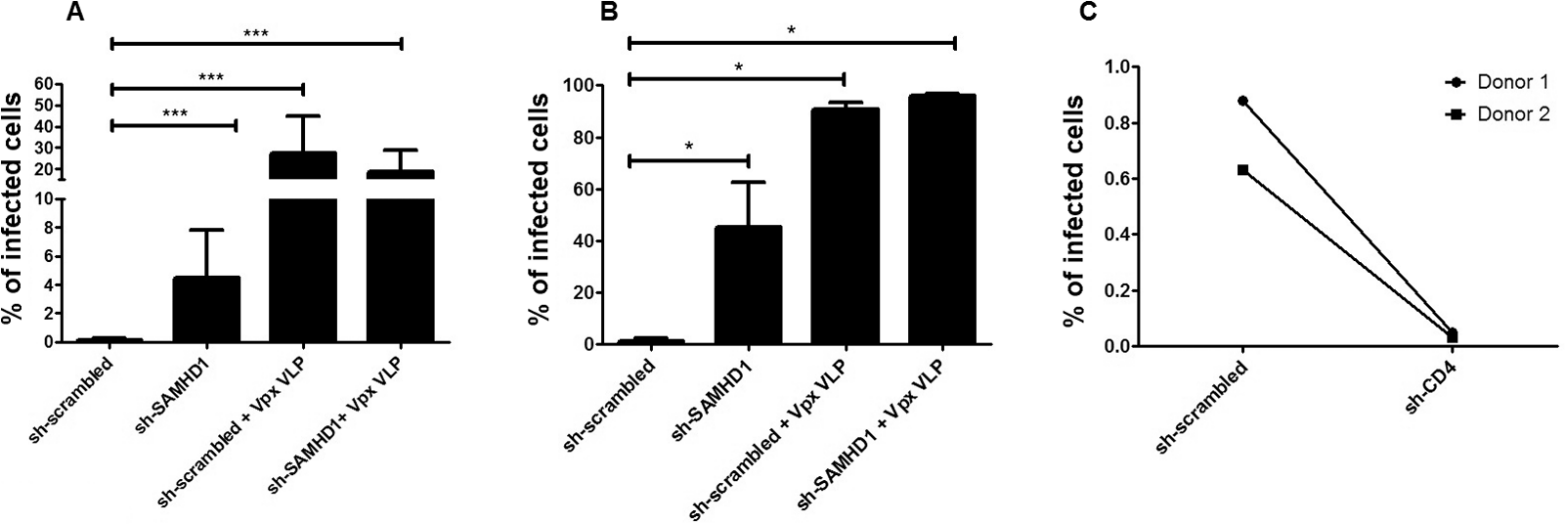
HIV infection of transduced MDDCs demonstrates functional effect on HIV infection. Five days post-transduction with lentiviral vectors ± Vpx VLP as shown, MDDCs were infected with non-pseudotyped (A) or VSV envelope-pseudotyped (B) HIV-1 engineered to express eGFP. Graph bars represent percentage of cells expressing eGFP encoded by HIV on day 3 post-infection. Error bars represent standard deviation among 9 (A) or 4 (B) donors. p values: *p ≤ 0.0286, ***p ≤ 0.0004. (C) MDDCs transduced with scrambled shRNA or CD4 targeting shRNA (but not eGFP) expressing lentiviral vectors, were infected on day 5 post-transduction with HIV engineered to express eGFP. Graph shows day 3 infection rates in MDDCs obtained from two donors.

In order to demonstrate functional effect of SAMHD1 down-regulation and to determine whether transduced MDDCs are infectable with HIV, we infected the lentivirally-transduced cells with a replication-competent HIV-1 five days post-lentiviral transduction in the presence of viral replication inhibitor ritonavir. To do so, we used a HIV NL4-3 virus, characterized by a complete HIV genome engineered to express eGFP as part of a bi-cistronic eGFP-IRES-Nef mRNA. By this set-up, the infection rate could accurately be measured three days post-infection. As expected, infection rates were boosted by SAMHD1 knock-down in MDDCs compared to scrambled control, most evident with Vesicular Stomatitis Virus envelope (VSV)-pseudotyped virus (Fig 6A and 6B). In line with the complete depletion of SAMHD1 observed in Vpx-loaded VLP-treated MDDCs, infection was highest for these cells, exceeding 90%. Conversely, shRNA-mediated down-regulation of HIV entry receptor CD4, resulted in reduced number of infected cells compared to control transduced MDDCs (Fig 6C).

## Discussion

Lentiviral gene delivery is the method of choice when stability of transgene expression in the absence of toxicity is needed and therefore it is an excellent tool for triggering shRNA interference in target cells. For MDDCs, expression of a transgene or shRNA sequence integrated into the host genome via lentiviral transduction has been problematic to date. Ideally over 95% of cells should express the transgene to avoid the need for further selection steps that may require cell sorters or time consuming drug-based selection, which by itself can affect function and phenotype of the cells. In the past, transduction with high MOI was the only way to achieve such high efficiencies. Unfortunately, in part due to the need to concentrate the virus prior to transduction, the use of high MOI results in maturation of MDDCs compromising their potential therapeutic use [7], or can even be toxic reducing MDDCs survival. Here we present an optimization of the transduction protocol, allowing over 95% of MDDCs transduction without the need for high MOI or addition of Vpx-loaded VLPs. Interestingly, comparable transduction efficiency was observed when monocytes were isolated with positive and negative microbead-based selection methods. Breckpot et al., clearly demonstrated [24] that signaling through CD14 receptor upon positive monocyte selection with anti-CD14 antibody coated microbeads facilitated lentiviral transduction compared to adhesion-based purification method. However, the protocol used in that study was optimized for transduction performed on day 3 post monocyte isolation. In the hereby protocol, monocytes are transduced 24 hours post isolation. Since it has been previously shown, that transduction early after isolation is beneficial, possibly it is also independent of selection method (i.e. CD14 stimulation) because the cells have not yet acquired the restrictive phenotype. It would be worthwhile to investigate whether that restriction is SAMHD1-mediated and if so, whether it depends on its levels and/or phosphorylation status. Isolation method might prove important depending on the downstream use of transduced MDDCs. For example, magnetic microbeads can interfere with electron microscopy. With presented protocol, both positively and negatively selected monocytes can be transduced comparably. Given the high efficiency of the process, there is no need for marker-based selection. The limited amount of vector used ensures preservation of the immature phenotype as shown, does not compromise viability of MDDCs and does not require pre-concentration of the viral stocks. We have not observed any impact of fetal calf serum origin (manufacturer) on transduction efficiency. The method was functionally validated by transducing cells with shRNA sequences targeting several MDDC expressed genes what demonstrated versatile, functional down-regulation on protein level as shown for CD4, SAMHD1 and DC-SIGN. Experiments with cells down-regulated for a well-known HIV restriction factor SAMHD1 were designed in order to simultaneously demonstrate two phenomena. First the sh-SAMHD1-transduced cells were more susceptible to HIV-1 infection compared to the scrambled shRNA control (35-fold on average). Such a functional effect of SAMHD1 down-regulation in primary MDDCs has not been reported with Vpx-free shRNA transduction in the past and provides further validation of the proposed method. In parallel, the cells were transduced with the same vectors in presence of Vpx-loaded VLP. As expected, addition of Vpx resulted in almost complete degradation of SAMHD1 in the scrambled shRNA control as well as sh-SAMHD1 expressing cells. Vpx is known to degrade SAMHD1 [10, 11] and allows for cells to be transduced with lentiviral vectors in low MOI without induction of MDDC maturation [9]. Degradation of SAMHD1 explains the difference with MDDCs transduced with sh-SAMHD1 alone, since the shRNA only blocks the production of newly synthetized SAMHD1, whereas in presence of Vpx an immediate degradation of the protein occurs. When we challenged those cells with VSV-pseudotyped HIV-1 virus in presence of a replication inhibitor, nearly 100% of cells got infected, demonstrating the remarkably efficient interference of Vpx with restriction of HIV by MDDCs. Recent reports describing SAMHD1 involvement in vaccinia and herpes simplex virus 1 infection [25, 26] show this interference not to be exclusive for retroviruses. Therefore, to minimize potential infection risks, the use of Vpx-loaded VLP must be avoided if one wants to deliver clinical grade manipulated DCs. Further on, especially in the context of HIV-1 research, use of Vpx might obscure the readout of experiments. Manel et al. have demonstrated that Vpx-mediated relieve of restriction in MDDCs leads to viral sensing and interferon secretion [27]. Useful in certain experimental approaches, such a dramatic change in HIV-host cell interaction imposes an additional level of complexity in an already artificial *in vitro* model. For the same reason, Vpx-assisted lentiviral transduction in dendritic-to-T cell HIV transmission studies should be performed with extra care. DCs are normally resistant to HIV-1 infection [28], but their inflammatory response to the virus in presence of Vpx was shown to highly activate surrounding T cells [27]. Our results showing a functional effect on HIV infection upon down-regulation of viral receptor CD4 and restriction factor SAMHD1 demonstrate that HIV studies can be performed with shRNA transduced MDDCs without the need for Vpx complementation. Recent data suggests that even HIV-2, naturally equipped with Vpx protein, did not evolve to overcome restriction of infection by DCs [29]. This underscores the treatment of DCs with Vpx to be biologically artificial. As probably many other aspects of the biological effects of Vpx are unknown, data obtained from experiments where Vpx is used should be interpreted with caution.

Expression of eGFP by the transduced MDDCs demonstrates that with the protocol presented here, biological applications are not limited to gene knock-down, but include expression of ectopic proteins. This opens the avenue for applications in cell-based cancer gene therapy, where lentiviral introduction of tumor associated antigens in MDDCs would prime other cells of the immune system to elicit anti-tumor responses [2, 30]. In such setting, transduction enhancement by Vpx pre-treatment of MDDCs would bring along additional safety issues. Therefore the method presented for Vpx-independent transduction and shRNA-mediated gene knock-down in MDDCs could be of value in both research and gene therapy.]

## Supporting Information

S1 Fig Highly efficient, Vpx-independent transduction is non-toxic and reproducible among tested donors. (A) The graph depicts number of cells/ml of culture on day 5 post transduction. (B) Transduction efficiency of sh-scrambled eGFP transduced MDDCs as shown in panel A.
(TIF)

S2 Fig Transduction efficiency is independent of isolation method. Monocytes isolated by positive (A) or negative (B) selection were transduced with scrambled shRNA and eGFP encoding vector, and analyzed by flow cytometry on day 5 post-transduction. Left panels show not transduced control, right panels transduced cells. Numbers indicate percentage of eGFP positive cells.
(TIF)

S3 Fig High transduction efficiency is independent of the serum used. Monocytes were transduced with scrambled shRNA and eGFP encoding vector and analyzed by flow cytometry on day 5 post-transduction. Throughout the culture, cells were maintained in medium supplemented with fetal calf serum obtained from indicated suppliers. Upper panels show not transduced control, lower panels transduced cells. Numbers indicate percentage of eGFP positive cells.
(TIF)

S4 Fig shRNA-mediated DC-SIGN down-regulation. MDDCs were transduced with scrambled shRNA or DC-SIGN targeting shRNA expressing lentiviral vectors. On day 5 post-transduction DC-SIGN surface levels were evaluated by flow cytometry. Dot plots show surface DC-SIGN expression gated on live (PI negative) cell population.
(TIF)
